# Intranodal Palisaded Myofibroblastoma in a Submandibular Lymph Node

**DOI:** 10.1155/2017/7121485

**Published:** 2017-10-26

**Authors:** Leila Bouhajja, Raja Jouini, Olfa Khayat, Wafa Koubâa, Chiraz Mbarek, Ehsen Ben Brahim, Achraf Chedly-Debbiche

**Affiliations:** ^1^Department of Pathology, Habib Thameur Hospital, University of Tunis El Manar, Tunis, Tunisia; ^2^Department of Otolaryngology, Habib Thameur Hospital, University of Tunis El Manar, Tunis, Tunisia

## Abstract

Intranodal palisaded myofibroblastoma (IPM), also known as “intranodal hemorrhagic spindle cell tumor with amianthoid fibers,” is a rare benign mesenchymal tumor originating from smooth muscle cells and myofibroblasts, often with the presence of amianthoid fibers. Usually IPM affects inguinal lymph nodes, but three cases have been described in the submandibular and cervical lymph nodes. We report a new case of a 44-year-old women with submandibular mass. Cervical ultrasound showed a suspect right submandibular adenomegaly. The patient underwent an excision of the submandibular mass. Histological features of the tumor include an encapsulated fusocellular proliferation, with nuclear palisading, amianthoid fibers, hemosiderin pigment, and extravasated erythrocytes. In the light of these results, we made the diagnosis of IPM. No recurrence was found 5 years after surgery.

## 1. Introduction

Intranodal palisaded myofibroblastoma (IPM), also known as “intranodal hemorrhagic spindle cell tumor with amianthoid fibers,” is a benign mesenchymal tumor of the lymph node originating from smooth muscle cells and myofibroblasts often with the presence of amianthoid fibers [1, 2]. Eighty-nine cases of IPM have been reported in the literature since its first description [3]. Usually, IPM arises within inguinal lymph nodes, but three cases have been described in submandibular and cervical lymph nodes [4, 5]. Its unique microscopic, macroscopic, and immunohistochemical features differentiate it from other intranodal mesenchymal tumors.

In this study, we report a new case of submandibular IPM.

## 2. Case Report

**Figure 1:**
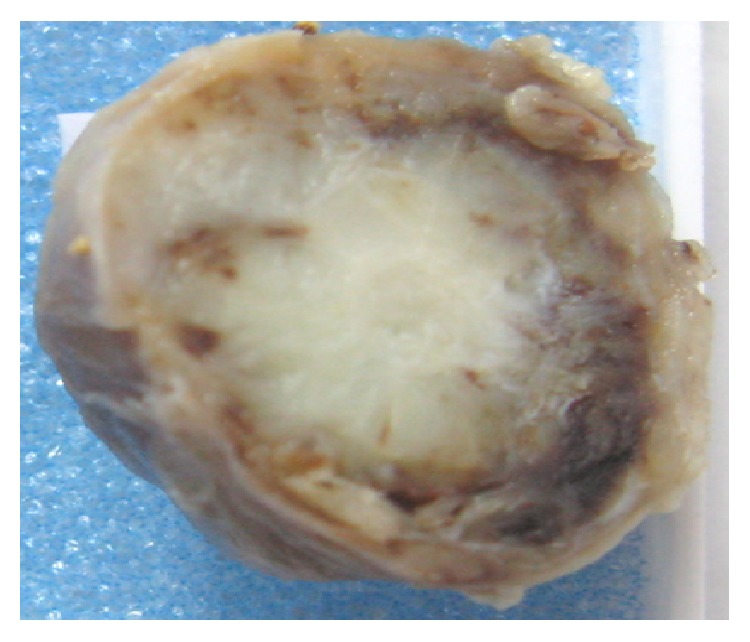
Grossly, the tumor had a pearly-white appearance and was well circumscribed, focally calcified with hemorrhagic alterations.

**Figure 2:**
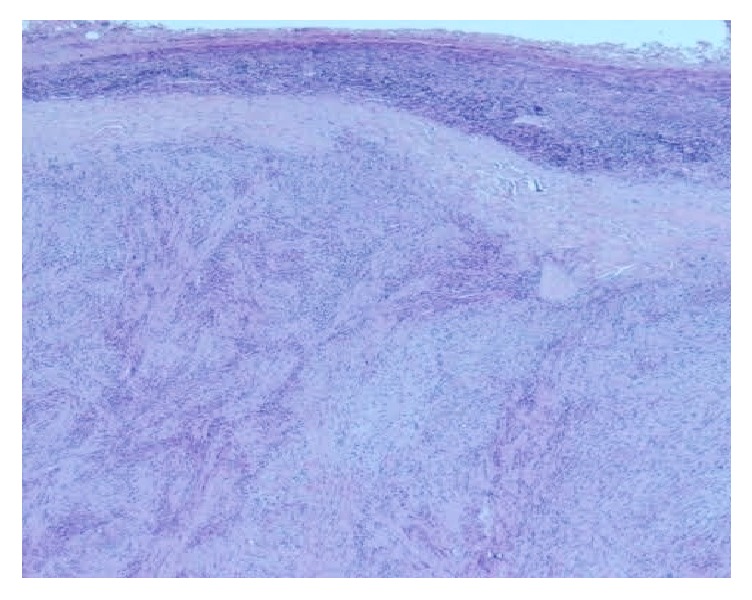
Fusocellular proliferation encapsulated by a thick fibrous capsule compressing the nodal tissue (HE, 100x).

**Figure 3:**
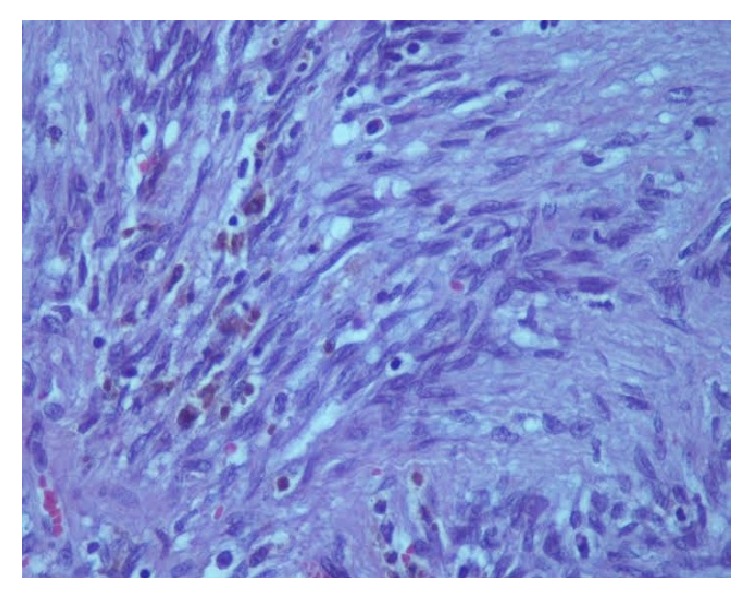
The lesion contains hemosiderin pigments (HE, 400x).

**Figure 4:**
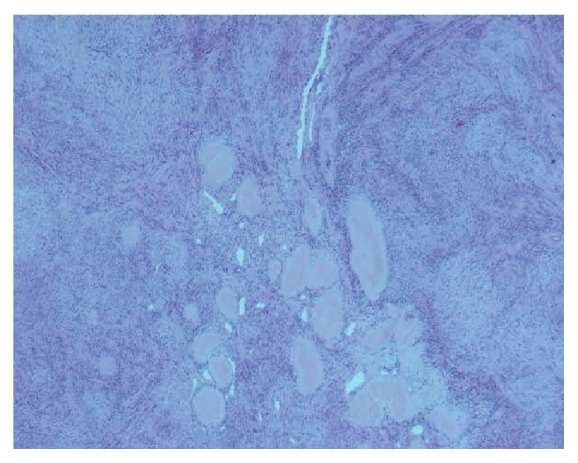
Acellular material accumulation with homogeneous eosinophilic appearance called “amianthoid fibers” (HE, 100x).

A 44-year-old-women presented with a 5-year history of a growing right submandibular swelling and 2-year history of anterior basicervical swelling without dysphagia, dysphonia, or dyspnea. On physical examination, the basicervical swelling consisted in a goiter that predominates in the right lobe. The submandibular mass was firm, well circumscribed, and painless measuring 3 cm in greatest diameter. The cervical echography showed a heterogeneous multinodular goiter and a suspect right submandibular adenomegaly. The thyroid function (TSH, FT4) was normal. The patient had a total thyroidectomy and an excision of the submandibular mass. Grossly, the submandibular lesion, 3 × 2 × 1 cm in size, was well circumscribed, firm, and focally calcified and had a pearly-white appearance with multifocal hemorrhagic areas on the cut surface (Figure 1). The mass was fixed in 10% formalin, embedded in paraffin, and stained with hematoxylin and eosin. Microscopic examination revealed a fusocellular proliferation encapsulated by a thick fibrous capsule (Figure 2). The cells were spindle shaped with abundant eosinophilic cytoplasm and elongated nuclei without mitoses or atypia. The cells were arranged in short bundles and were winded in a storiform appearance. Nuclear palisading was focally observed and gave it a schwannoma appearance. Otherwise, hemorrhagic alterations and hemosiderin pigment were present (Figure 3). A striking feature was the presence of satellite areas of homogeneous and eosinophilic deposits identical to those previously described as “amianthoid fibers” (Figure 4). Masson trichrome confirmed that these ones were collagen deposits. A rim of lymph node was observed at the periphery of the tumor.

**Figure 5:**
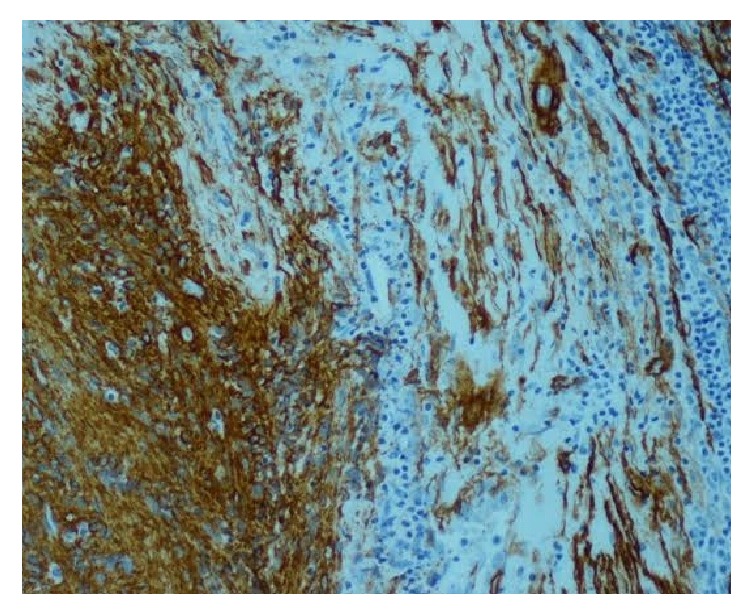
The spindle cells were diffusely immunoreactive for SMA.

Immunohistochemical stains showed a strong immunoreactivity for vimentin and Smooth Muscle Actin (SMA) (Figure 5). The tumor cells were negative for desmin, S-100 protein, CD34, CD31, CD117, CD68, EMA, and keratin. Tumor cells did not express HMB-45. Immunostaining for cyclin D1 and HHV8 was negative in these cells with a low proliferating index of Ki67 (<1%). In the light of these results, the case was diagnosed as intranodal palisaded myofibroblastoma.

The patient was doing well at 5-year follow-up, with no evidence of recurrence.

## 3. Discussion

IPM is uncommon. About 89 cases have been reported in the literature [3]. It is a benign mesenchymal neoplasm characterized by intranodal proliferation of spindle cells with smooth muscle differentiation, often with the presence of amianthoid fibers [1, 2]. IPM commonly affects the second to eighth decade, with a peak incidence in the group between 40 and 60 years of age, but the occurrence of this tumor in an infant has also been reported [6, 7]. It is more common in men with a male to female ratio of 1.5 and is not specific to any race [2]. Usually IPM arises within inguinal lymph nodes. Rare cases affect cervical, axillary, and submandibular lymph nodes like our case [4, 6, 8]. Macroscopically, the cut surface of the lymph node is firm, has a gray white colour, and shows irregular hemorrhagic areas. Microscopic features of IPM, as fascicles of spindle cells with nuclear palisading, amianthoid fibers, hemosiderin pigment, and extravasated erythrocytes, were observed in our case. Nuclear atypia is absent and mitoses are rare. Immunohistochemically, the spindle cells are positive for SMA and vimentin. The most important differencial diagnoses of IPM are schwannoma and Kaposi's sarcomas. Schwannomas contain spindle cells with nuclear palisading but, normally, schwannoma does not exist in lymph nodes. IPM contains extravasated erythrocytes and hemosiderin pigments among the spindle cells, with nuclear palisading and amianthoid fibers that are not observed in Kaposi's sarcoma. Moreover, IPM has to be distinguished from other soft tissue tumors such as intranodal leiomyoma, leiomyosarcoma, dendritic cell sarcoma, spindle cell melanoma, and inflammatory myofibroblastic tumor [2, 9]. The clinical history, the physical examination, and the typical histological characteristics help in the correct diagnosis of IPM. The histogenesis of IPM remains controversial. It probably arises from myofibroblasts or from differentiated smooth muscle cells of the walls of blood vessels in the lymph node [4, 9]. IPM has recently been shown to have a strong expression for cyclin D1 and a low proliferating index of Ki-67. Some authors demonstrated cyclin D1 expression in 50% of spindle cells in these lesions. The positive staining for cyclin D1 suggests the possible role of the cell cycle regulatory genes in the pathogenesis of IPM [10]. IPM has been associated with infections from Epstein Bar Virus (EBV) and Human Herpes Virus-8 (HHV-8) [11]. In our case, there was no evidence of HHV-8 and EBV infection, and cyclin D1 overexpression was not found. Excellent prognosis after surgical treatment and no malignant transformation have been reported [11]. Local recurrence was reported in only two cases [4].

## 4. Conclusion

Intranodal palisaded myofibroblastoma (IPM) is a rare benign mesenchymal tumor. It is a problem of differential diagnosis mainly with benign intranodal schwannoma. The treatment is based on complete excision of the lesion. Its evolution is benign. The histogenesis of this entity remains controversial. Further studies of large tumor series using molecular techniques would elucidate the pathogenesis of this lesion.
